# Complete Mitogenome Sequencing, Annotation, and Phylogeny of *Grateloupia turuturu*, a Red Alga with Intronic *cox1* Gene

**DOI:** 10.3390/life13081642

**Published:** 2023-07-28

**Authors:** Maheshkumar Prakash Patil, Jong-Oh Kim, Young-Ryun Kim, Seokjin Yoon, Kyunghoi Kim

**Affiliations:** 1 Industry-University Cooperation Foundation, Pukyong National University, 45 Yongso-ro, Nam-gu, Busan 48513, Republic of Korea; 2 Department of Microbiology, Pukyong National University, 45 Yongso-ro, Nam-gu, Busan 48513, Republic of Korea; 3 School of Marine and Fisheries Life Science, Pukyong National University, 45 Yongso-ro, Nam-gu, Busan 48513, Republic of Korea; 4 Marine Eco-Technology Institute, Busan 48520, Republic of Korea; 5 Dokdo Fisheries Research Center, National Institute of Fisheries Science, Pohang 37709, Republic of Korea; 6 Department of Ocean Engineering, Pukyong National University, 45 Yongso-ro, Nam-gu, Busan 48513, Republic of Korea

**Keywords:** *cox1* intron, *Grateloupia turuturu*, intronic ORF, red algae, Halymeniales, phylogenetic analysis, mitochondrial DNA

## Abstract

The mitochondrial genome (mitogenome) is essential for identifying species and tracing genetic variation, gene patterns, and evolutionary studies. Here, the mitogenome of *Grateloupia turuturu* was sequenced on the Illumina sequencing platform. This circular mitogenome (28,265 bp) contains 49 genes, including three rRNAs, twenty transfer RNAs (tRNAs), and twenty-six protein-coding genes (PCGs). Nucleotide composition indicates biased AT (68.8%) content. A Group II intronic sequence was identified between two exons of the *cox1* gene, and this sequence comprises an open reading frame (ORF) that encodes a hypothetical protein. The gene content, annotation, and genetic makeup are identical to those of Halymeniaceae members. The complete mitogenome sequences of the *Grateloupia* and *Polyopes* species were used in a phylogenetic analysis, which revealed that these two genera are monophyletic and that *G. turuturu* and *G. elliptica* are closely related. This newly constructed mitogenome will help us better understand the general trends in the development of *cox1* introns in Halymeniaceae, as well as the evolution of red algal mitogenomes within the Rhodophyta and among diverse algal species.

## 1. Introduction

Rhodophyta algae (red algae) are an evolutionarily significant eukaryotic lineage which inhabit marine and freshwater. Rhodophyta species are mostly multicellular, photoautotrophic, and abundant in marine habitats (around 98%) and rare in freshwater, with a few rare terrestrial or sub-aerial representatives [1]. The red alga have photosynthetic pigments, chlorophylls *a* and *d*, and characteristic red colors due to the phycoerythrin pigment. In the evolutionary sense, red algae are plant-like because they have a single shared parent with green algae (Chlorophyta) and higher plants (Embryophyta) [2,3]. Rhodophytes are divided into seven classes with around 7538 species, and among them, the Florideophyceae class possesses the maximum number of species (7141), which are mostly marine, multicellular algae including seaweeds [4].

A marine-habituated red macroalga, *Grateloupia turuturu* (Y. Yamada, 1941), classified under the phylum, Rhodophyta; class, Florideophyceae; subclass, Rhodymeniophycidae; order: Halymeniales; family, Halymeniaceae; and genus, Grateloupia [5]. There are 69 *Grateloupia* species that have been classified, and 36 species are still unclassified, and out of these, only 5 complete *Grateloupia* mitochondrial genomes are available on the National Center for Biotechnology Information (NCBI) website (https://www.ncbi.nlm.nih.gov, accessed on 15 June 2023). These species are found all around the world, including in the Atlantic islands, Caribbean islands, Europe, North and South America, Africa, Asia, Australia, and New Zealand [4]. Furthermore, there are 227 species listed under the family, Halymeniaceae, but to date, only 7 species (*Grateloupia angusta*, *G*. *cornea*, *G*. *elliptica*, *G*. *filicina*, *G*. *taiwanensis*, *Polyopes affinis,* and *P*. *lancifolius*) with complete mitogenome have been reported [6,7,8,9,10,11], as listed in Table 1.

Relatively little is known about the mitogenome of Rhodophytes, and due to advancements in software and molecular technologies, more and more detailed studies are being reported. In fact, red algal mitogenomes are more complete than previously reported [12], and it has also been reported that red algae, *Strylonematophyceae*, contain multiple minicircular mitochondrial genomes that encode one or two genes [13]. These studies are made possible by applying bundles of software tools. The red algal mitogenomes have less molecular weight than other algae, and because of their maternal inheritance, they are a useful tool for evolutionary and phylogenetic studies. In addition, mitogenomes have a specific sequence that gives reliable data for studying the gene order, makeup, contents, and secondary structures of the encoded RNA [14,15], and it is also useful for making molecular kits (barcoding markers) for economically important species identification [16]. The *Grateloupia* species contain a characteristic intronic *cox1* gene (Table 1), and such features are useful to understand evolutionary and phylogenetic studies [3,6,7,8,9,17]. Algae mitogenomes consist of introns in the genic region, tandem repeats, and large intergenic repeats, which create challenges for assembling complete circular mitogenomes [15] but due to revolutionary advances in sequencing technologies and bioinformatics tools, such issues can be overcome. So, utilizing modern, next-generation sequencing methods and bioinformatics tools, we provide here the full mitochondrial genome of red algae as well as a phylogenetic relationship based on the complete mitogenome sequence.

In this study, we used de novo assembly on the Illumina platform to sequence the complete circular mitogenome of *G. turuturu*. Gene annotation, genetic makeup, and gene order were confirmed using several bioinformatics tools and phylogenetic studies based on complete mitogenome sequencing. This study’s data were submitted to the NCBI GenBank and will be useful for future research on the evolution and phylogeny of red algae species.

## 2. Materials and Methods

### 2.1. Sample Collection and DNA Isolation

A deep-sea diver from the Marine Eco-Technology Institute in Busan, South Korea, collected *Grateloupia turuturu* from the coast of Gijang (35°28′ N, 129°25′ E) and then deposited it there under the voucher number PU-T01-S-MA-04 (contact person: Dr. Young-Ryun Kim, yykim@marine-eco.co.kr). Total DNA was extracted using the QIAGEN DNEasy Blood and Tissue Kit (QIAGEN, Hilden, Germany) as per the manufacturer’s protocol, and the purity and concentration of DNA were confirmed via a NanoDrop spectrophotometer (Thermo Fisher Scientific D1000, Waltham, MA, USA). Purified total genomic DNA samples were kept at −20 °C until required.

### 2.2. Whole Genome Sequencing

*G. turuturu* genome was sequenced using the Illumina Platform (Illumina Inc., San Diego, CA, USA). The library preparation and sequencing processes were carried out by the Macrogen Company in Daejeon, South Korea. Sequencing libraries were prepared using the TrueSeq Nano DNA Kit according to the manufacturer’s protocol, and sequencing was performed on the Illumina HiSeq 2500 Platform in paired-end 150 bp mode. Before downstream analysis, raw data initially underwent quality checks to obtain clean reads. The low-quality bases (phred quality score, Q < 20), empty reads, and Illumina adapters were removed to mitigate the analytical bias by Trimmomatic [18]. After filtering, 12,903,396 total reads (GC = 40.05%, Q20 = 99.26%) were produced from a total of 14,873,050 raw reads (GC = 40.23%, Q20 = 97.33%). The overall quality of the produced sequencing reads was verified using FastQC v0.11.5 (Babraham Institute, Bioinformatics) [19], and mitogenome de novo assembly was finished using various *k*-mers [20] and the SPAdes v3.13.0 program [21].

### 2.3. Mitogenome Assembly and Annotation

Mold/Protozoan Mitochondrial was selected for the genetic code; red algae belonging to the Florideophyceae and Bangiophyceae classes have demonstrated this method of codon translation [3,6,7,8,9,10,11]. The mitogenome annotation was performed using the MFannot tool (https://megasun.bch.umontreal.ca/apps/mfannot/, accessed on 10 May 2023) with genetic code 4 (Protozoan Mitochondrial Code) [22]. The final annotation was checked and verified using ORFfinder (https://www.ncbi.nlm.nih.gov/orffinder/, accessed on 10 May 2023), and predicted open reading frames (ORFs) were checked manually and annotated accordingly. Protein-coding genes (PCGs) were verified with previously sequenced red algal mitogenomes by BLAST homology searches against the NCBI database [23]. Transfer RNA was identified using tRNAscan-SE v2.0 (http://lowelab.ucsc.edu/tRNAscan-SE/, accessed on 10 May 2023) with the default setting (with Model: Mold & Protozoa Mito) [24]. The tRNA genes, rRNA genes, and introns were identified using RNAweasel (https://megasun.bch.umontreal.ca/apps/rnaweasel/, accessed on 10 May 2023) [25]. Tandem Repeat Finder (TRF) was used to identify and annotate the repeats in the mitogenome sequence [26]. The assembled contig was analyzed for identification by querying BlastN [23,27] for known red algae mitogenomes and comparing mitogenome sizes.

A physical map of the mitogenome was designed with OrganellarGenomeDRAW v. 1.3.1 (https://chlorobox.mpimp-golm.mpg.de/OGDraw.html, accessed on 15 June 2023) [28]. The nucleotide composition of the mitogenome was estimated using MEGA11 v.11.2.8 [29]. Codon usage and relatively synonymous codon usage (RSCU) for collected ORFs of PCGs were analyzed by the Sequence Manipulation Suite (SMS) tool with genetic code 4 (http://www.bioinformatics.org/sms2/codon_usage.html, accessed on 10 May 2023) [30]. The following formula was used to calculate the asymmetric base composition of the mitochondrial genome: GC − skew = [G − C]/[G + C] and AT − skew = [A − T]/[A + T] [31].

### 2.4. Phylogenetic Analysis

The phylogenetic tree was made by using the complete circular mitogenome sequences of eight red algae from the family Halymeniaceae (Table 1) and one alga from the family Glaucocystaceae (*Glaucocystis nostochinearum*, GenBank accession number HQ908425) as an out-group member. All mitogenomes utilized in this investigation were obtained from the NCBI GenBank. The dataset was initially processed by ClustalW for multiple sequence alignment in MEGA11 [32]. Multiple sequenced aligned datasets were used to generate a maximum-likelihood phylogenetic tree using the Tamaru–Nei model and 1000 replicated bootstraps in MEGA11 with the default parameters [29,33].

### 2.5. Data Availability

The mitogenome sequence and related data were submitted to the NCBI GenBank (http://www.ncbi.nlm.nih.gov/, accessed on 12 May 2023 and 16 June 2023). The complete mitogenome sequence is available for the public under the accession number OQ972988, along with associated data including Sequence Read Archive (SRA), BioProject, and BioSample with the assigned numbers PRJNA984428, SAMN35767756, and SRR24947511, respectively.

## 3. Results and Discussion

### 3.1. Genome Size and Organization

**Figure 1:**
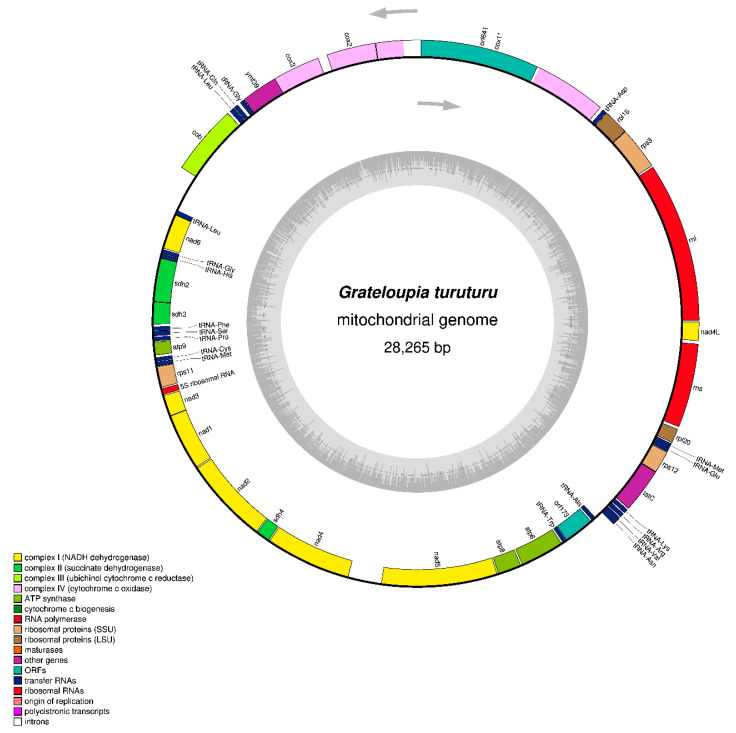
Gene map of the *Grateloupia turuturu* (OQ972988) mitochondrial genome. Different categories of genes are represented by abbreviations and arrows outside and inside the circle, which indicates the direction of gene transcription. A gene (*cox1*) containing group II introns is denoted with an asterisks. The map was drawn using OrganellarGenomeDRAW (https://chlorobox.mpimp-golm.mpg.de/OGDraw.html, accessed on 15 June 2023).

The contig with a length of 28,265 bp was identified as the mitochondrial genome; based on BlastN analysis, it matches the reference species of *Grateloupia*, and the mitogenome size is comparable to that of other red algal mitogenomes (Table 1). The mitogenome sequence of *Grateloupia turuturu* is available in GenBank with accession number OQ972988. The complete circular mitogenome map with gene arrangement is shown in Figure 1. The contig is 28,265 bp long and is composed of A = 36.1%, T = 32.7%, G = 16.1%, and C = 15.5%, with a bias of 68.8% A + T contents. The *G. turuturu* mitogenome contains 3 rRNA, 20 tRNA, and 26 PCGs (including intronic and hypothetical protein genes), including 14 respiratory chain subunits (complexes 1–4), four ATP synthase subunits (complex 5), two each of LSU and SSU ribosomal proteins, one independent protein translocase (*tatC*), and two hypothetical protein genes (*orf641* and *orf173*). Among these genes, 24 (12 PCGs, 10 tRNAs, and 2 rRNAs genes) are found on the heavy strand (H-strand), while the rest (14 PCGs, 10 tRNA, and 1 rRNA gene) are found on the light strand (L-strand). The positive AT skew (0.049) and GC skew (0.032) were observed in this study with the presence of more A and G than T and C, respectively (Table 1). In comparison to *Grateloupia* [6,7,8,9] and *Polyopes* [10,11] species with complete mitogenome features, the mitogenome of *G. turuturu* demonstrates no significant gene losses; however, *G. elliptica* (OP479979) [7] has closer mitogenome features in terms of nucleotide composition, bias AT content, and gene compositions. In Halymeniales, the typical complete mitogenome was circular and approximately 25 to 30 kb in length with correspondingly conserved gene content, which encoded 24 PCGs (excluding intronic and hypothetical genes), 2–3 rRNAs, and 18–23 tRNAs with A + T bias nucleotides (Table 1) [6,7,8,9,10,11].

### 3.2. Protein-Coding Gene Features

The PCG area, which included intronic and hypothetical genes, made up 71.53% of the *G. turuturu* mitogenome and was 20,220 base pairs long. *nad5* is the longest PCG with 1998 bp, while *atp9* is the smallest with 231 bp. Each PCG was initiated by a canonical ATG codon, except for *tatC*, which was initiated by a TTG codon (Table 2). Similar results have been demonstrated in *G. cornea* (OQ910480), *G. elliptica* [7], and *P. affinis* [10]. Furthermore, out of 26 PCGs, 21 terminated with the TAA codon, except 5 PCGs (*sdh2*, *cox2*, *atp8*, *atp6*, and *rps11*) which terminated with the TAG codon, which was typical for *Grateloupia* [6,7,8,9] and *P. lancifolius* [11]. The *G. turuturu* mitogenome was analyzed for intergenic nucleotide, and it was noted that junctions of three gene pairs have an overlap; 1 bp each between *trnL* (number 2)–*nad6* and *trnH*–*sdh2*, and 51 bp between *cox3*–*ymf39*. Furthermore, the intergenic gaps differ from 1 bp to 650 bp in length, with the longest gap of 650 bp between *cob*–*trnL* (number 2) (Table 2).

Analysis of the complete mitogenome sequence of *G. turuturu* revealed the presence of a group II intron segment (position: 5042–7298) between two exons of *cox1*, which encodes an ORF (*orf641*; position: 5084–7009). Hypothetical genes *orf641* and *orf173* (Table 2) with an unknown function were identified and encoded hypothetical proteins. Similar outcomes have been documented for all *G. angusta, G. cornea, G. elliptica, G. filicina,* and *G. taiwanensis*, but not for *P. affinis* and *P. lancifolius* (Table 1) [6,7,8,9,10,11]. Group II intronic *cox1* and *trnI* are the unique features of red algal mitogenomes [12,13,15]. The *ymf39* gene is transcribed between *cox3* and *trnG* genes in the mitogenome of *G. turuturu* and encodes an ATP synthase B chain precursor. Similar annotations were reported for *Grateloupia* species [6,7,8,9] and *P. lancifolius* [11], but the annotation for *P. affinis* is *atp4* [10]. In recent studies, a reanalysis of red mitogenome sequences revealed that the *atp4* gene was annotated with the name, *ymf39*, instead of its original name [12,34]. It is suggested that the *ymf39* ORF encodes for ATP synthase chain b; therefore, Florideophyceae mitogenome annotation should change *ymf39* to *atp4* to avoid further confusion [35]. Likewise, the conserved sequence of PCG encodes a sec-independent protein translocase protein annotated (identified) with *tatC* [7,10] and *secY* [6,8,9,11] names within the species of Florideophyceae. In the review of red algae, scientists noted that *secY* is not found in the algal mitogenome and recommended that the *secY* annotation be changed to *tatC* [12,34,35,36]. The *rpl20* gene is located between *rrs* and *trnM* in the *G*. *turuturu* mitogenome (Figure 1). The gene content of the mitogenomes of *G. turuturu* and other species of *Grateloupia* [6,7,8,9] and *Polyopes* [10,11] is identical, with the exception of the absence of *rpl20* in *G*. *cornea* (OQ910480).

Codon usage analysis of the 26 PCGs of the *G. turuturu* mitogenome (intronic and hypothetical ORFs included) showed that 6714 amino acid triplets were expressed (Table 3), not including stop codons. Leucine (N = 990, 14.74%) and cysteine (N = 85, 1.27%) are the most and least abundant amino acids, respectively. Furthermore, the most frequently used codons in PCGs include TTA (N = 544, 8.10%, Leu), TTT (N = 507, 7.55%, Phe), ATT (N = 390, 5.80%, Ile), AAA (N = 291, 4.33%, Lys), and GTT (N = 199, 2.96%, Val). The present study results are in line with the mitogenome of *G. cornea* (OQ910480).

### 3.3. Ribosomal RNA and Transfer RNA

The mitogenome of *G. turuturu* consists of three rRNAs (Table 4): two small subunits (*rns* = 1367 bp and *rrn5* = 108 bp) and one large subunit (*rnl* = 2596 bp). Two rRNAs (*rnl* and *rns*) are transcribed on the H-strand and separated by the *nad4L* gene. However, the *rrn5* gene is located between *nad3* and *rps11* and is transcribed on the L-strand. Similar annotations have been reported for the mitogenomes of *Grateloupia* [6,7,8,9] and *Polyopes* [10,11] species, except for the absence of *rrn5* in the mitogenomes of *G. angusta* [6], *G. filicina* [8], *G. taiwanensis* [9], and *P. lancifolius* [11].

Twenty tRNAs were identified in the mitogenome of *G. turuturu* (Table 2), accounting for 5.23% (1495 bp) of the total length of the mitogenome; the length of individual tRNAs ranges from 70 (*trnQ*-TTG) to 85 bp (*trnL*-TAA and *trnS*-TGA). In addition, an equal number of tRNAs were transcribed on both strands (H- and L-strands). The number of tRNAs ranged from 18 to 24, with small variations in the tRNA gene content among the Halymeniaceae family members shown in Table 4. The mitogenome of *G. turuturu* contains double copies of three tRNA (*trnG*, *trnL*, and *trnM*), of which two tRNA (*trnG*, *trnL*) use different anticodons. Additionally, the mitogenome lacks *trnI* and *trnY*, and there is no intronic tRNAs. The *trnI* is the intronic tRNA gene, present in the *G. angusta* [6], *G. filicina* [8], *G. taiwanensis* [9], and *P. lancifolius* [11]. At least two copies of *trnM*-CAT (except three copies in *G. angusta* [6]) were present in all examined species, suggesting a major role for this tRNAs in Halymeniaceae mitogenomes. It should be noted that *trnR*-TCT (Arg) and *trnS*-GCT (Ser) were absent in *G. turuturu* although they could be found in other known Rhodophyte mitogenomes.

### 3.4. Phylogenetic Analysis

**Figure 2:**
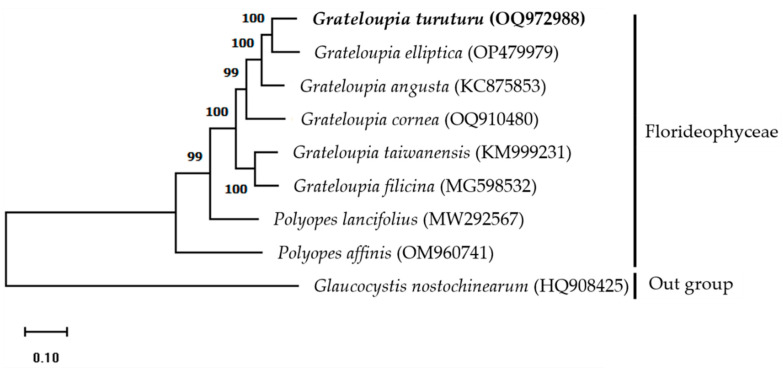
Maximum-likelihood (ML) phylogenetic tree based on complete mitogenome sequences indicating the relationship between red algae (family Halymeniaceae). A alga from the Glaucocystaceae family was used as an outgroup member. Bootstrap support values are indicated at nodes. NCBI GenBank accession numbers are listed next to the corresponding species names.

The mitogenome maximum-likelihood (ML) phylogenetic tree was constructed using a complete mitogenome sequence of Halymeniaceae members obtained from GenBank and *G. nostochinearum* as an out-group member (Figure 2). Results indicate that *G. turuturu* is positioned next to *G. elliptica*, suggesting a close relationship. All members of the Halymeniaceae family are monophyletic, and the clade is strongly supported (99–100 percent bootstrap values). The ML phylogenetic relationships based on complete mitogenome sequences [7,10] and PCGs [8,11] indicate that the *Grateloupia* (intronic *cox1* gene-containing) and *Polyopes* species are monophyletic. Our phylogenetic analysis results are consistent with previous studies. The findings of this study will be helpful for taxonomic and phylogenetic research on red algae.

## 4. Conclusions

In this study, we reported the complete mitogenome of *G. turuturu* (OQ972988), which is circular, 28,265 bp in length, with AT bias (68.8%) composition, encoding 49 genes including 26 PCGs, 20 tRNA, and 3 rRNA genes. This mitogenome contains the intronic cox1 gene with functional ORF similar to those in *G. angusta*, *G. cornea*, *G. elliptica*, *G. filicina,* and *G. taiwanensis*. The *G. turuturu* mitogenome lacks the intronic tRNA-Ile (*trnI*) that is present in the mitogenomes of *G. angusta*, *G. filicina*, *G. taiwanensis,* and *P. lancifolius*. We may learn more about the evolution of red algal mitogenomes within the Rhodophyta species and across different algal species with the help of this newly constructed mitogenome, as well as about the general patterns in the development of *cox1* introns in Halymeniaceae.

## Figures and Tables

**Table 1:** An overview of the complete mitogenomes utilized in this study.

Algae	*G. turuturu*	*G. angusta*	*G. cornea*	*G. elliptica*	*G. filicina*	*G. taiwanensis*	*P. affinis*	*P. lancifolius*
GenBank no.	OQ972988	KC875853	OQ910480	OP479979	MG598532	KM999231	OM960741	MW292567
Size (bp)	28,265	27,943	30,595	28,503	29,274	28,906	25,988	26,132
Nucleotide composition								
A (%)	36.1	36.7	35.3	36.2	35.6	36.0	37.9	36.1
T (%)	32.7	33.1	31.6	32.6	32.4	32.6	34.6	32.9
G (%)	16.1	15.4	16.8	15.9	16.4	16.2	14.3	15.8
C (%)	15.1	14.7	16.3	15.3	15.5	15.3	13.3	15.2
AT (%)	68.8	69.8	66.9	68.8	68.0	68.6	72.5	69.0
GC (%)	31.2	30.1	33.1	31.2	31.9	31.5	27.6	31.0
AT-Skew	0.049	0.052	0.055	0.052	0.047	0.050	0.046	0.046
GC-Skew	0.032	0.023	0.015	0.019	0.028	0.029	0.036	0.019
Group of genes (numbers)								
rRNA	3	2	3	3	2	2	3	2
tRNA	20	18	23	20	24	24	23	23
PCGs ^a^	26	26	25	26	26	26	25	25
Other features								
Intronic ORF	Yes	Yes	Yes	Yes	Yes	Yes	No	No
Intronic *cox1*	Yes	Yes	Yes	Yes	Yes	Yes	No	No
Intronic tRNA	No	Yes	No	No	Yes	Yes	No	Yes
Unique genes	*orf641*, *orf173*	Gang5, Gang35	*orf632*, *orf173*	*orf634*	*cox1*-intronic ORF, *orf174*	*cox1*-intronic ORF, *orf172*	*orf164*	*orf165*
Reference	In this study	[6]	-	[7]	[8]	[9]	[10]	[11]

Note: ^a^ Including the intronic ORF and hypothetical protein genes.

**Table 2:** Sequence characteristics of *G. turuturu* (OQ972988) mitochondrial genome.

Group	Group of Genes	Gene Name	Three Letter Code	Location		Size (bp)	No. of Amino Acid	Strand	Start Codon	Stop Codon	Anti-Codon	Intergenic Nucleotides ^a^
				Start	End							
rRNA	Large subunit of a ribosome	*rnl*	-	20	2615	2596	-	H	-	-	-	23
	Small subunit of a ribosome	*rrn5*	-	15281	15388	108	-	L	-	-	-	15
		*rns*	-	26545	27911	1367	-	H	-	-	-	48
tRNA	Transfer RNA genes	*trnD*	Asp	3760	3831	72	-	H	-	-	GTC	51
		*trnG*	Gly	10054	10128	75	-	H	-	-	TCC	49
		*trnQ*	Gln	10178	10249	70	-	H	-	-	TTG	7
		*trnL*	Leu	10257	10341	85	-	H	-	-	TAA	40
		*trnL*	Leu	12178	12259	82	-	L	-	-	TAG	−1
		*trnG*	Gly	12886	12957	72	-	L	-	-	GCC	5
		*trnH*	His	12963	13037	75	-	L	-	-	GTG	−1
		*trnF*	Phe	14214	14286	73	-	L	-	-	GAA	4
		*trnS*	Ser	14291	14375	85	-	L	-	-	TGA	15
		*trnP*	Pro	14391	14464	74	-	L	-	-	TGG	11
		*trnC*	Cys	14744	14814	71	-	L	-	-	GCA	7
		*trnM*	Met	14822	14895	74	-	L	-	-	CAT	4
		*trnW*	Trp	23819	23891	73	-	L	-	-	TCA	7
		*trnA*	Ala	24444	24518	75	-	L	-	-	TGC	140
		*trnN*	Asn	24659	24731	73	-	H	-	-	GTT	2
		*trnV*	Val	24734	24805	72	-	H	-	-	TAC	13
		*trnR*	Arg	24819	24893	75	-	H	-	-	ACG	17
		*trnK*	Lys	24911	24983	73	-	H	-	-	TTT	21
		*trnE*	Glu	26106	26178	73	-	H	-	-	TTC	3
		*trnM*	Met	26182	26254	73	-	H	-	-	CAT	15
CDS	NADH dehydrogenase subunits (complex 1)	*nad6*	-	12259	12867	609	202	L	ATG	TAA	-	18
		*nad3*	-	15404	15769	366	121	L	ATG	TAA	-	2
		*nad1*	-	15772	16752	981	326	L	ATG	TAA	-	18
		*nad2*	-	16771	18258	1488	495	L	ATG	TAA	-	14
		*nad4*	-	18529	20004	1476	491	L	ATG	TAA	-	477
		*nad5*	-	20582	22579	1998	665	L	ATG	TAA	-	18
		*nad4L*	-	27960	28265	306	101	H	ATG	TAA	-	19
	Succinate dehydrogenase (complex 2)	*sdh2*	-	13037	13789	753	250	L	ATG	TAG	-	1
		*sdh3*	-	13791	14174	384	127	L	ATG	TAA	-	39
		*sdh4*	-	18273	18512	240	79	L	ATG	TAA	-	16
	Apocytochrome b (complex 3)	*cob*	-	10382	11527	1146	381	H	ATG	TAA	-	650
	Cytochrome c oxidase (complex 4)	*cox1* ^ b^	-	3883	5041	1599	532	H	ATG	-	-	-
				7299	7738			H	-	TAA	-	3
		*cox2*	-	7742	8539	798	265	H	ATG	TAG	-	144
		*cox3*	-	8684	9502	819	272	H	ATG	TAA	-	−51
	ATP synthase (complex 5)	*ymf39*	-	9450	10049	600	199	H	ATG	TAA	-	4
		*atp9*	-	14476	14706	231	76	L	ATG	TAA	-	37
		*atp8*	-	22598	23008	411	136	L	ATG	TAG	-	24
		*atp6*	-	23033	23794	762	253	L	ATG	TAG	-	24
	SSU ribosomal proteins	*rps3*	-	2639	3334	696	231	H	ATG	TAA	-	2
		*rps11*	-	14900	15262	363	120	L	ATG	TAG	-	18
		*rps12*	-	25735	26100	366	121	H	ATG	TAA	-	5
	LSU ribosomal proteins	*rpl16*	-	3337	3753	417	138	H	ATG	TAA	-	6
		*rpl20*	-	26270	26503	234	77	H	ATG	TAA	-	41
	Independent protein translocase	*tatC*	-	25005	25733	729	242	H	TTG	TAA	-	1
	Hypothetical proteins	*orf641*	-	5084	7009	1926	641	H	ATG	TAA	-	289
		*orf173*	-	23899	24420	522	173	L	ATG	TAA	-	23

Note: ^a^ The number of nucleotides between the given and previous gene, with a negative value indicating an overlap; ^b^ *cox1* gene-exon number 1 (3883–5041), intron (5042–7298), and exon number 2 (7299–8539); H and L indicate that the genes are transcribed on the heavy and light strands, respectively.

**Table 3:** Codon usage of PCGs in the mitogenome of *G. turuturu* (OQ972988).

Amino Acids	Codon	Number	%	Fraction	Amino Acids	Codon	Number	%	Fraction	Amino Acids	Codon	Number	%	Fraction
Ala	GCG	42	0.626	0.12	Gly	GGT	139	2.070	0.39	Ser	AGT	122	1.817	0.23
	GCA	130	1.936	0.38		GGC	39	0.581	0.11		AGC	50	0.745	0.09
	GCT	150	2.234	0.44	His	CAT	100	1.489	0.74		TCG	41	0.611	0.08
	GCC	20	0.298	0.06		CAC	36	0.536	0.26		TCA	153	2.279	0.29
Arg	AGG	17	0.253	0.09	Ile	ATA	194	2.889	0.29		TCT	131	1.951	0.25
	AGA	57	0.849	0.30		ATT	390	5.809	0.59		TCC	37	0.551	0.07
	CGG	8	0.119	0.04		ATC	75	1.117	0.11	Thr	ACG	40	0.596	0.11
	CGA	33	0.492	0.17	Leu	TTG	122	1.817	0.12		ACA	111	1.653	0.31
	CGT	52	0.775	0.27		TTA	544	8.102	0.55		ACT	176	2.621	0.49
	CGC	26	0.387	0.13		CTG	35	0.521	0.04		ACC	35	0.521	0.10
Asn	AAT	214	3.187	0.69		CTA	117	1.743	0.12	Trp	TGG	28	0.417	0.20
	AAC	96	1.430	0.31		CTT	153	2.279	0.15		TGA	110	1.638	0.80
Asp	GAT	111	1.653	0.67		CTC	19	0.283	0.02	Tyr	TAT	169	2.517	0.60
	GAC	55	0.819	0.33	Lys	AAG	67	0.998	0.19		TAC	113	1.683	0.40
Cys	TGT	55	0.819	0.65		AAA	291	4.334	0.81	Val	GTG	38	0.566	0.09
	TGC	30	0.447	0.35	Met	ATG	166	2.472	1.00		GTA	140	2.085	0.34
Gln	CAG	35	0.521	0.18	Phe	TTT	507	7.551	0.84		GTT	199	2.964	0.48
	CAA	159	2.368	0.82		TTC	97	1.445	0.16		GTC	39	0.581	0.09
Glu	GAG	39	0.571	0.19	Pro	CCG	24	0.357	0.11	*	TAA	-	-	-
	GAA	165	2.458	0.81		CCA	73	1.087	0.33		TAG	-	-	-
Gly	GGG	36	0.536	0.10		CCT	104	1.549	0.48					
	GGA	143	2.130	0.40		CCC	17	0.253	0.08					

Note: Amino acids—three-letter code; %—Percentage of each amino acid specified by a given codon in the *G. turuturu* mitogenome; *—asterisks denote termination codons (excluded from analysis).

**Table 4:** Mitochondrial rRNA and tRNA in Halymeniaceae.

Algae	*G. turuturu* (OQ972988)	*G. angusta*(KC875853)	*G. cornea* (OQ910480)	*G. elliptica* (OP479979)	*G. filicina* (MG598532)	*G. taiwanensis* (KM999231)	*P. affinis* (OM960741)	*P. lancifolius* (MW292567)
rrn5	1	0	1	1	0	0	1	0
rns	1	1	1	1	1	1	1	1
rnl	1	1	1	1	1	1	1	1
*trnA* (TGC)	1	1	1	1	1	1	1	1
*trnC* (GCA)	1	1	1	1	1	1	1	1
*trnD* (GTC)	1	0	1	1	1	1	1	1
*trnE* (TTC)	1	1	1	1	1	1	1	1
*trnF* (GAA)	1	1	1	1	1	1	1	1
*trnG* (TCC)	1	1	1	1	1	1	1	1
*trnG* (GCC)	1	1	1	1	1	1	1	1
*trnH* (GTG)	1	0	1	1	1	1	1	1
*trnI* (GAT)	0	1	0	0	1	1	0	1
*trnK* (TTT)	1	1	1	1	1	1	1	1
*trnL* (TAA)	1	1	1	1	1	1	1	1
*trnL* (TAG)	1	0	1	1	1	1	1	1
*trnM* (CAT)	2	3	2	2	2	2	2	2
*trnN* (GTT)	1	1	1	1	1	1	1	1
*trnP* (TGG)	1	1	1	1	1	1	1	1
*trnQ* (TTG)	1	1	1	1	1	1	1	1
*trnR* (ACG)	1	1	1	1	1	1	1	1
*trnR* (TCT)	0	0	1	0	1	1	1	1
*trnS* (GCT)	0	0	1	0	1	1	1	0
*trnS* (TGA)	1	1	1	1	1	1	1	1
*trnV* (TAC)	1	1	1	1	1	1	1	1
*trnW* (TCA)	1	0	1	1	1	1	1	1
*trnY* (GTA)	0	0	1	0	1	1	1	1
Total tRNA	20	18	23	20	24	24	23	23
Ref.	In this study	[6]	-	[7]	[8]	[9]	[10]	[11]

## Data Availability

The mitogenome sequence data that support the findings of this study are openly available in GenBank of NCBI at https://www.ncbi.nlm.nih.gov/ under accession number OQ972988.
